# Efficient processing of top-k frequent spatial keyword queries

**DOI:** 10.1038/s41598-022-10648-4

**Published:** 2022-05-05

**Authors:** Tao Xu, Aopeng Xu, Joseph Mango, Pengfei Liu, Xiaqing Ma, Lei Zhang

**Affiliations:** 1 Henan Key Laboratory of Big Data Analysis and Processing, Henan University, Kaifeng, 475004 China; 2 School of Computer and Information Engineering, Henan University, Kaifeng, 475004 China; 3 Key Laboratory of Geographical Information Science, Ministry of Education, East China Normal University, Shanghai, 200241 China; 4 Key Research Institute of Yellow River Civilization and Sustainable Development, Henan University, Kaifeng, 475004 China; 5 Henan Technology Innovation Center of Spatio-Temporal Big Data, Henan University, Zhengzhou, 450046 China; 6 Henan Industrial Technology Academy of Spatio-Temporal Big Data, Henan University, Zhengzhou, 450046 China; 7 Department of Transportation and Geotechnical Engineering, University of Dar es Salaam, P.O. 35131 Dar es salaam, Tanzania

**Keywords:** Computer science, Information technology

## Abstract

The rapid popularization of high-speed mobile communication technology and the continuous development of mobile network devices have given spatial textual big data (STBD) new dimensions due to their ability to record geographical objects from multiple sources and with complex attributes. Data mining from spatial textual datasets has become a meaningful study. As a popular topic for STBD, the top-k spatial keyword query has been developed in various forms to deal with different retrievals requirements. However, previous research focused mainly on indexing locational attributes and retrievals of few target attributes, and these correlations between large numbers of the textual attributes have not been fully studied and demonstrated. To further explore interrelated-knowledge in the textual attributes, this paper defines the top-k frequent spatial keyword query (tfSKQ) and proposes a novel hybrid index structure, named RCL-tree, based on the concept lattice theory. We also develop the tfSKQ algorithms to retrieve the most frequent and nearest spatial objects in STBD. One existing method and two baseline algorithms are implemented, and a series of experiments are carried out using real datasets to evaluate its performance. Results demonstrated the effectiveness and efficiency of the proposed RCL-tree in tfSKQ with the complex spatial multi keyword query conditions.

## Introduction

Advancements of mobile networks and intelligent terminal devices have led spatial textual big data (STBD) to increase tremendously and cause many challenges of their efficient retrievals. In general, STBD comprises spatial location information, e.g. latitude and longitude, textual keyword information of spatial objects, e.g. name, address, etc., and the rich domain of knowledge to integrate spatial texts and the posed locations around the querying points. Top-k spatial keyword query (TkSKQ) is currently a common way to STBD retrieval. It takes spatial ranges and textual keywords as query parameters to retrieve the STBD set and returns top k eligible objects. This type of query is used mainly in business information benefiting from the Location-based Devices and Services (LBS). Therefore, on this basis, it’s evident that to ensure and secure good accessibility to the STBD, the retrieval quality and efficiency of the TkSKQ algorithms are the keys in this domain.

**Figure 1:**
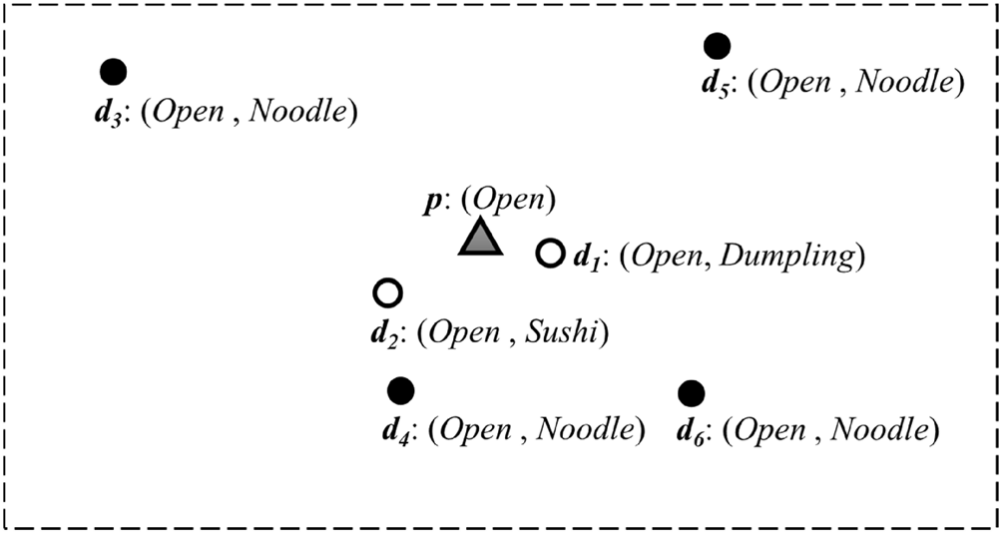
An example of TkSKQ for “*open*” restaurants.

Most of the existing TkSKQ algorithms^1–8^ focus on user preferences to match the degree of spatial and textual keywords between individual spatial objects and search targets. Since they ignore regional features of the search space, some questions about the similarity of spatial objects, e.g. “What are the most frequent items?”, are not answered directly. This scenario can be well explained using a query example for restaurants, shown in Fig. 1, where dots represent restaurant POIs with some textual features. A query employs "*Open*" as a textual keyword to retrieve the two restaurants closest to the query point *p*. If considerations are vested only to the spatial proximity and textual keyword consistency, "*Dumpling*" (*d*_1_) and "*Sushi*" (*d*_2_) will be provided as retrieval results, while the regional feature of search space is "*Noodle*" determined by *d*_*3*_, *d*_*4*_, *d*_*5*_, *d*_*6*_. That means, the most popular "*Open*" restaurants with “*Noodle*” aren’t recommended to the user if that user is not in the appropriate spatial location. Therefore, in such circumstances, the aggregation of features of the search space should be considered, and further analysis of frequent items of TkSKQ results need to be explored.

Several studies for frequent item analysis are done, and their more details are presented in “Related Work” section. Most of them are the hybrid index structure, they employ table-based structure^9–14^ to maintain the textual keyword information to achieve top-k frequent spatial keyword queries. Since, in STDB, the textual keywords of spatial object are diverse and complex, the number of frequent features of them is often more than data itself, and the retrieval of frequent features by above table-based index structure still needs a high cost. Which makes it challenging to retrieve the top-k frequent items from STBD, especially in the scenario of multi textual keywords.

In this paper, we propose a top-k frequent Spatial Keyword Query (tfSKQ) algorithm to explore the similarity of spatial textual objects and retrieve the frequent items from STBD. Compared with TkSKQ, tfSKQ gives more preferences on textual relevancy than it, and pays more attention to the exploration of the typical or popular textual keywords from STDB. With the given spatial and textual query conditions, the proposed tfSKQ algorithm can find the k frequent items efficiently for STBD. Along to this target, we also develop a novel hybrid index structure, *R-tree concept lattice* (named RCL-tree), to support the proposed tfSKQ algorithm. RCL-tree is a hybrid index structure that includes R-tree^15^ structure and concept lattice^16^ structures to maintain spatial objects with multi textual keywords. Spatial information is maintained by R-tree, and textual keywords information is organized by concept lattice. Concept lattice is a partial order set of concepts generalized from data records. The concept defines the common keywords of a group of data records, and can directly represent the frequent features of data. Therefore, we employ R-tree to store spatial information of spatial objects, and each node of R-tree links to a distinct concept lattice that organizes the textual information of spatial objects. In fact, we employ concept lattice only when the number of objects in the R-tree node is within a given range. It can effectively reduce the time cost of RCL-tree initialization since the construction of concept lattice is time consuming.

The proposed RCL-tree and tfSKQ algorithms are experimented with the real data set from yelp.com to verify their performance and usability. And some comparison evaluations are conducted with one existing method^10^ (called δSTLs) and two baseline methods based on Apriori^17^ algorithm and FP-Growth^18^ algorithm (called A-frequent and F-frequent respectively). Comparison results demonstrate that the proposed RCL-tree and tfSKQ algorithms have the strong applicability to STBD and have the best retrieval efficiency than others in tfSKQ with multi query keywords.

The main contributions of this research are as follows: (1) We propose a hybrid index structure, *R-tree Concept Lattice* (named RCL-tree), to index STBD. It can not only be used to index spatial information and support TkSKQ but also maintain the correlation of textual information and answer tfSKQ with more efficiency and accuracy. (2) We develop a top-k frequent spatial keyword query (tfSKQ) algorithm to retrieve the frequent items in search space based on the RCL-tree. It aims to find the top k frequent items in the search space with given query conditions about spatial proximity, text consistency and feature frequency. (3) We conduct a series of experiments using two real datasets to evaluate the effectiveness of the proposed RCL-tree and the tfSKQ algorithm.

## Related work

Top-k spatial keyword query (TkSKQ) is a hot research topic in recent years. Most of the existing research works employ specific hybrid index structures to index spatial information and textual information, respectively, and design retrieval algorithms to answer TkSKQ. For spatial information indexing, R-tree^15^ and its variants are the most common ones^1,2,4,6,19–22^. Cary et al*.*^2^ propose a hybrid Spatial-Keyword Index (SKI) for spatial textual data; it combines R-tree with inverted indices to maintain spatial and textual information of the spatial object. De Felipe et al*.*^4^ proposed Information Retrieval R-tree (I^2^R-tree) with R-tree and bitmap structure. Cong et al*.*^1^ and Li et al*.*^6^ combine R-tree with inverted files to develop a hybrid index structure IR-tree for spatial object dataset. And Rocha-Junior et al*.*^21^ and Attique et al*.*^22^ tries to develop the hybrid index structures including R-tree, inverted files, and others to answer TkSKQ in Road network. In addition, quadtree^23^ is also employed for indexing spatial textual data. For example, SFC-QUAD^3^ combines quadtree with inverted files, IL-Quadtree^8^ use the space-filling curve technique to construct quadtree for each keyword to organize, e.g. spatio textual objects effectively. On the other hand, grid structure is also used for a spatial keyword query. For example, Khodaei et al*.*^5^ and Vaid et al*.*^7^ combined a grid structure with inverted files to index spatial objects. Li et al*.*^19^ proposes a Topology‐based Mixed Index Structure (TMIS) to index network‐constrained trajectories for connectivity‐based queries. Another study by Xu et al*.*^24^ employed a cube structure and B-tree structures to answer queries of the spatial–temporal textual big data in road networks. These combinations imply that the hybrid model of index structure with spatial index and textual index is suitable for retrieving spatial textual data.

Other efforts also develop separate index structure to answer TkSKQ. Chen et al.^25^ proposes a series of algorithms for spatial data query in geographic search engines. They try to index a large number of web pages by the strategies of text-first and location-first respectively, and develop some corresponding TkSKQ algorithms. Kwon et al.^14^ propose an efficient separate index method called Rank-Aware Separate Index Method (RASIM) which includes a tree spatial index structure and some individual inverted indexes for each keyword. Due to RASIM organizes spatial objects as a rank-aware group according to the spatial proximity, and the text relevancy of spatial objects is stored in the rank-aware group, RASIM supports top-k pruning and efficient merging at the same time, its retrieval efficiency is obviously better than that of IR-tree^1^. However, because the RASIM stores all of individual inverted indexes into a single file, theoretically, too many keywords will make a “big” index file and affect the retrieval efficiency.

With the advent of spatial textual big data, its hugeness and complexity make many difficulties for TkSKQ, not only for the efficiency of TkSKQ but also for the quality of retrieval results, requiring more consideration. Some efforts^11,13,26–30^ used the pivot based hierarchical method to explore the relationship between textual keywords of spatial objects to answer semantic frequent TkSKQ. Other efforts made so far focused to maintain users’ social relationships and respond to social-aware TkSKQ and some typical queries configured for such purposes include: the geo-social skyline keyword query (GSSK)^12^, social TkSKQ^31^, social-aware top-k spatial keyword (SkSK) query^9^, socio-spatial skyline query (SSSQ)^32^, and top-k frequent spatiotemporal terms (kFST) query^10^. In these studies, some invert table-based index structures are employed to organize textual keywords of spatial objects, and the generalized knowledge, i.e., frequent items, contained in a group of spatial textual data can be presented. However, such invert table-based index structure cannot cope well with the significant increase and complexity of STBD^33^, especially in the complex spatial multi keyword query, because there are usually the demands of large filtering and traversal operations to extract frequent items from table-based structure. Therefore, it is very necessary to generalize the textual keywords of spatial objects to facilitate the extraction of frequent items in STBD.

Meanwhile, some conceptual inference based methods have been successfully used to further aggregate the results of TkSKQ and mine the implicit intentions in textual keywords of spatial data. For example, Xu et al*.*^34^ proposes a conceptual inference-based method (CISK) to generate some concepts by considering typicality, granularity and spatial distribution, and link them with the hypernym–hyponym relationships in knowledge graphs. And the user-preferred spatial objects are ranked and recommended. In addition, Schwering and Raubal^35^ employ geospatial concept model to generalize spatial objects and employ semantic similarity of concepts to measure the spatial relations. Moreover, as a suitable model for presenting the hierarchy and relationship of concepts, concept lattice, proposed by Wille et al*.*^16^, are also employed to deal with spatial data analysis. Such as, Kainz et al*.*^36^ employs ordered sets and lattice structures to describe the spatial relationship of spatial data, Chen et al*.*^37^ proposes a concept lattice-based method to mine spatial association rules, Tripathy et al*.*^38^ employs a lattice structure to achieve data analysis in Spatial Data Warehouse, And Wu et al*.*^39^ a fuzzy formal concept analysis-based approach to uncovering the spatial hierarchies among vague places, etc.

Concept lattice is an efficient knowledge mining tool. It maintains a poset of concepts and can be represented by a Hasse graph, in which each node is a concept, to reveal the relationship between objects and attributes. It has been widely used in information retrieval^40^, software engineering^41^, recommendation system^42^, and knowledge discovery^43^, etc. A concept in concept lattice is the explicit results of data aggregation, and can be represented as a set of spatial objects with several common keywords. The number of spatial objects in a concept directly represents the frequency of keyword combinations of this concept. Clearly, employing concept lattice to maintain textual information must facilitate to achieve frequent items retrieval. However, according to our review, no research results on frequent item retrieval of spatial data based on concept lattices have been published.

In this paper, we attempt to employ concept lattice for the first time to retrieve the top k frequent items in a search space and achieve the tfSKQ for STBD. Different with TkSKQ, tfSKQ attempt to discover the frequency of the textual keywords of spatial objects while TkSKQ only retrieve the spatial objects that match the query keywords. To achieve tfSKQ, we propose a novel hybrid index structure called RCL-tree by deploying a R-tree structure and some concept lattice structures to maintain spatial information and textual keywords information of STBD, respectively. And a one-to-one mapping existed between partial R-tree nodes and concept lattices. To achieve it, a threshold for R-tree nodes capacity is defined to determine which R-tree nodes need to link with concept lattice. In concept lattice, the concept node includes two parts: the extent, i.e. spatial objects, and the intent, i.e. the common keywords of these spatial objects, the frequency of concept is the number of extent (spatial objects) in the concept, and the frequent items are the intent of concept. Then, the frequent items can be retrieved by traversing concept lattices and the tfSKQ for STBD can be answered by the proposed RCL-tree and tfSKQ algorithms.

## Methodology

**Figure 2:**
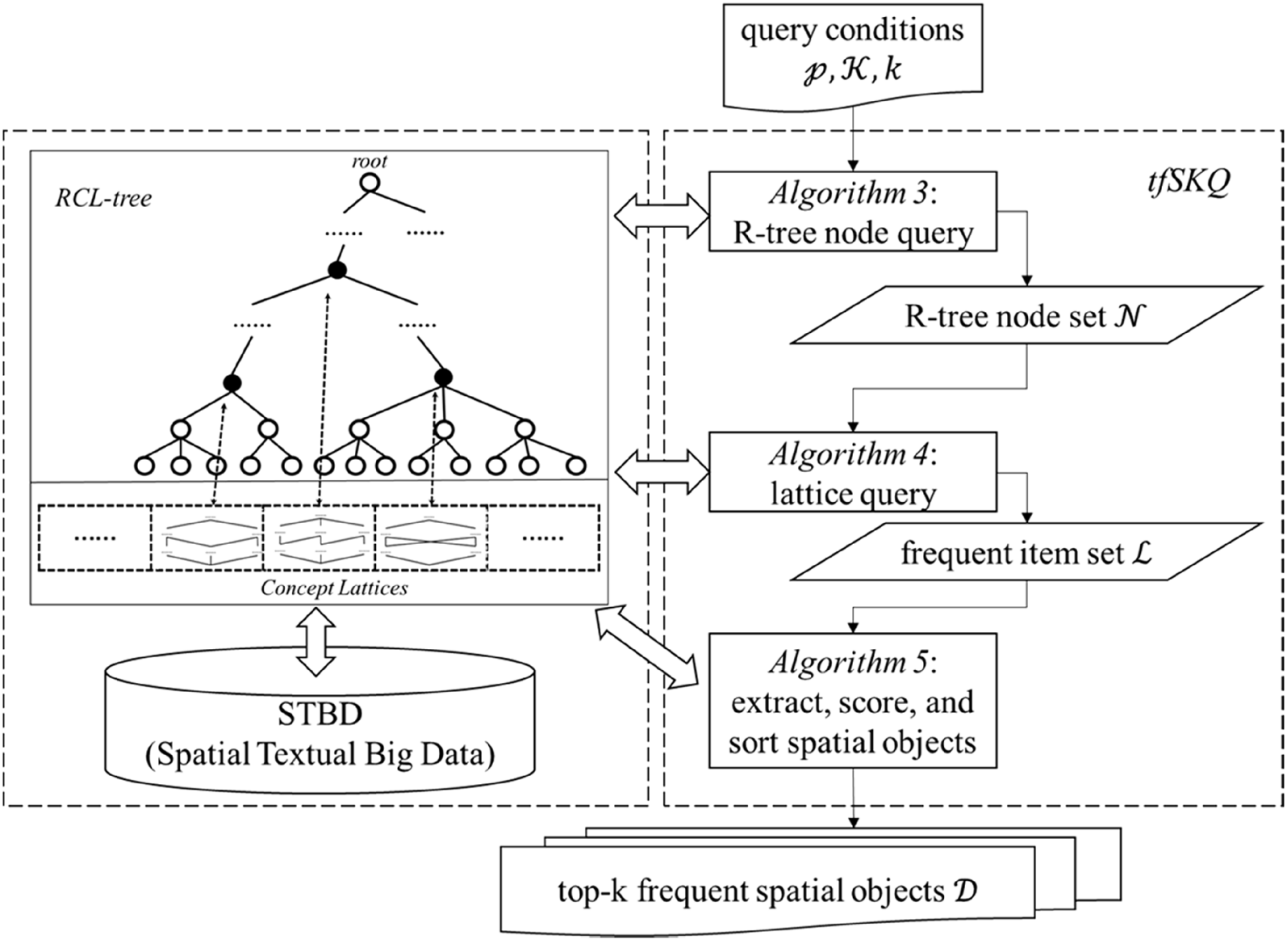
Schematic overview of RCL-tree.

The proposed RCL-tree is a hybrid index structure for STBD, it employs R-tree to index the spatial location of spatial objects and employs some concept lattice structures linked with R-tree nodes to model frequent patterns of spatial objects. Based on RCL-tree, the proposed tfSKQ algorithm is developed to answer the top k most frequent spatial objects with the query conditions: location point, textual keywords. The schematic overview of the RCL-tree and the tfSKQ algorithm are shown in Fig. 2. The RCL-tree consists of a tree structure and a concept lattice list, black nodes represent specific tree nodes linked with a concept lattice structure of list. The tfSKQ algorithm, with the given query conditions, first retrieves R-tree nodes set ${\mathcal{D}}$ adjacent to the target location from tree structure in *Algorithm 3*, and gets the corresponding concept lattice structure set ${\mathcal{L}}$ in *Algorithm 4*, then traverses ${\mathcal{L}}$ to retrieve the k spatial objects with most frequent features in *Algorithm 5*. The detailed processes are as follows.

### Index structure

RCL-tree is a hybrid index structure designed for answering tfSKQ in STBD. Some formal definitions are as follows.

#### Spatial textual big data

Let ${\mathbb{D}} = \{ d_{i} |1 \le i\}$ be a spatial textual big data (STBD) set, where $d_{i} = \left\langle {id,{ }p,K} \right\rangle$ is the ith spatial textual data record, $p$ is the spatial information, i.e. spatial position coordinates, and $K = \{ \left\langle {k_{1} ,k_{2} , \ldots ,k_{j} } \right\rangle {|}k_{j} \in \left\{ {0,1} \right\},1 \le j\}$ is the textual keyword set, $k_{j}$ is the jth textual keyword of $d_{i}$ and its value is 0 or 1.

#### R-tree

Is a popular spatial index structure proposed by Guttman in 1984. It employs the Minimum Bounding Rectangle (MBR) of multi spatial granularity hierarchy to organize spatial objects and achieve query in logarithmic level efficiency. Let ${\mathbb{R}} = \{ r,\theta ,{ }\left\langle {n_{1} , n_{2} , \ldots ,n_{i} } \right\rangle |1 \le i\}$ be a R-tree, where $r$ is the root of R-tree, $\theta = \left[ {\theta_{min} ,\theta_{max} } \right]$ is the range of node entries, $n_{i} = \left\langle {id, mbr,level, pn, cns,dn, ds} \right\rangle$ is the i*th* node and each node contains the node identification, $id$, the extent of MBR, $mbr$, the level of node, $level$, the root node has the highest level, the parent node, $pn$, the child nodes, ${\text{c}}ns$, whose size is limited by $\theta$, the number of spatial objects included in MBR, $dn$, and the data set, $ds \subset {\mathbb{D}}$. In R-tree, each $d_{i}$ in ${\mathbb{D}}$ can be organized based on spatial position coordinates, i.e. $d_{i} .p$.

Concept Lattice^16^ is a very important data analysis tool and is good at discovering and extracting from complex datasets. It derives from the structured data set (also called “**formal context**”), and represents concepts and their partial order relationships. Let $F = \left( {D,K,I} \right)$ be a formal context, where $D$ is the object set, $K$ is the attribute set, and $I \subseteq D \times K$ is the relationship between $D$ and $K$, $\left( {d,k} \right) \in I$ or $dIk$ represents object *d* has attribute *k*.

1 $$f\left( X \right) = \{ k|k \in K,\forall d \in X,X \subseteq D,\left( {d,k} \right) \in I\}$$

2 $$g\left( Y \right) = \{ d|d \in D,\forall k \in Y,Y \subseteq K,\left( {d,k} \right) \in I\}$$

Moreover, two operators ${\varvec{f}}$ and ${\varvec{g}}$ are defined in Eqs. (1) and (2) to formalize the relationship between $D$ and $K$. The $f$ operator is to solve the common attributes of an object set in a formal context. In Eq. (1), $f\left( X \right) = \left\{ k \right\}$ represents the common attribute set of the object set $X$ is $\left\{ k \right\}$, i.e. each object in the object set $X$ has the attribute set $\left\{ k \right\}$.

Based on $f$ and $g$, the common features of objects in formal context can be presented, and the object set can be abstracted as concept with some explicit attributes.

#### Concept

Let $C = \left\langle {X,Y} \right|X \subseteq D,Y \subseteq K, f\left( X \right) = Y,g\left( Y \right) = X >$ be a concept, where $X$ is called the **extent** of the $C$ concept and $Y$ is called the **intent** of the $C$ concept, $f\left( X \right) = Y$ and $g\left( Y \right) = X$ represent the extent $X$ and intent $Y$ of the $C$ concept satisfy both the $f$ and $g$ operators.

Let $\le$ be a partial order relationship between two concepts, $C_{1} = \left( {X_{1} ,Y_{1} } \right)$, $C_{2} = \left( {X_{2} ,Y_{2} } \right)$, then $C_{1} \le C_{2}$ meet Eq. (3). It represents $C_{1}$ is the sub concept of $C_{2}$, and $C_{2}$ is the super concept of $C_{1}$.

3 $$C_{1} \le C_{2} \Leftrightarrow X_{1} \subseteq X_{2} \left( { \Leftrightarrow Y_{2} \subseteq Y_{1} } \right)$$

#### Concept lattice

Based on $\le$, concepts extracted from $F$ can be related, and the hierarchy order of them can be established. Let $L = \left\{ {nid,F,{\mathcal{C}}, \le } \right\}$ be a concept lattice, where $nid$ is the identification of node in ${\mathbb{R}}$, $F$ is a formal context, ${\mathcal{C}}$ is a concept set, and $\le$ is a hierarchy order of ${\mathcal{C}}$. Note that a concept lattice $L$ links to a R-tree node where $L.F = {\mathbb{R}}.{\text{n}}_{i} .ds$, i.e. $L.F$ is the data set of R-tree node.

#### RCL-tree

Let ${\mathbb{I}} = \left\langle {{\mathbb{R}},\user2{ }{\mathbb{L}}} \right\rangle$ be a RCL-tree index structure, where ${\mathbb{R}}$ is a R-tree structure, ${\mathbb{L}} = \{ L_{1} ,L_{2} , \ldots ,L_{i} |1 \le i,L_{i} .F.size \in \delta \}$ is a concept lattice set. and $\delta = \left[ {\delta_{min} ,\delta_{max} } \right]$ is a threshold range of data volume. Concept lattices only link to partial R-tree nodes, and $\delta$ is a limitation to determine which R-tree nodes need to be linked to concept lattices. We choice R-tree nodes whose data volume is in the given range $\delta$, and set their data as a formal context to build concept lattice structure. Since the concept lattice is a complete set of a formal context, it contains all the relationships between spatial objects and textual attributes, the initialization complexity of concept lattice is directly proportional to the data volume, and the maintenance of multi concept lattices is complex and time-consuming. In addition, according to the retrieval mechanism of R-tree, the search of spatial proximity objects starts from tree root to leaf nodes, and the number of objects in these searched nodes decreases gradually. Then, some intermediate tree nodes must cover the target searching space and enough query candidates can be supply by one or more nodes. Therefore, we want to set the $\delta$ to limit the creation of concept lattice only for some appropriate intermediate tree nodes. It can not only reduce the initialization cost but also improve the efficiency of retrieval. In this way, STBD can be maintained, and tfSKQ can be achieved.

**Figure 3:**
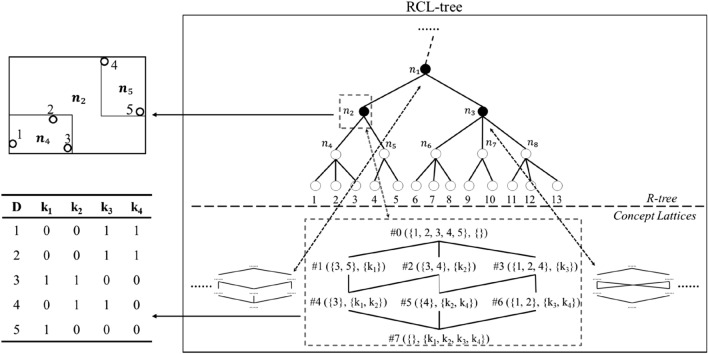
An example of RCL-tree.

Figure 3 shows an example of RCL-tree structure with $\delta = \left[ {5,13} \right]$, We highlight three R-tree nodes, $n_{1}$ and $n_{2}$ and $n_{3}$, met $\delta$ and built concept lattice for each one. $n_{2}$ includes a formal context with 5 data records and 4 keyword attributes, and the linked concept lattice consists of 8 concepts. In this concept lattice, it is easy to see that the extent of each concept is a frequent item for its intent keywords group, and with given spatial and textual keywords query conditions, the tfSKQ can be achieved by traversing all concept lattice at once.

### Initialization algorithm

The initialization algorithm of the proposed RCL-tree is given in Algorithm 1. Its inputs are a STBD set, ${\mathbb{D}}$, the threshold of R-tree node entries, $\theta$, and the threshold of the data volume of concept lattice $\delta$. Its output is an RCL-tree index structure.

As shown in Algorithm 1, R-tree is built based on the spatial information of spatial objects $d_{i} .p$ by traversing ${\mathbb{D}}$ in lines 1 to 3; then, every node of R-tree is checked by $\delta$, formal context and concept lattice structure are built with the textual keywords of spatial objects $d_{i} .K$, and concept lattice set ${\mathbb{L}}$ is generated in lines 4 to 12; finally, RCL-tree index structure ${\mathbb{I}}$ is finished by combining ${\mathbb{R}}$ and ${\mathbb{L}}$.
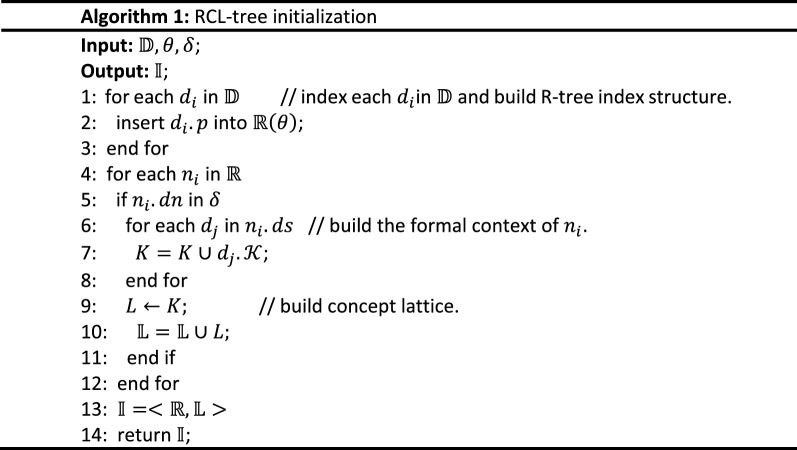


### The top-k frequent spatial keyword query (tfSKQ) algorithm

The target of the tfSKQ is to find out the k most frequent items that meet the spatial and keyword query conditions. Different from the TkSKQ (Top-k Spatial Keyword Query) method which outputs the query objects sorting by the spatial distance between object and query point, the tfSKQ method takes the frequency of objects as the primary criterion for filtering and sorting query results. With the support of RCL-Tree, the main idea of tfSKQ is to traverse the R-tree structure in RCL-tree to find out the tree nodes that contain spatial objects that are close to the given spatial query point, and then, based on the keyword frequency and spatial proximity of spatial objects, the top k most frequent and nearest spatial objects are retrieval from the concept lattices linked with tree nodes.

tfSKQ can be defined as $Q_{f}$ and let ${\mathcal{D}} = Q_{f} \left( {{\mathcalligra{p}},{\mathcal{K}}, k,{\mathbb{I}}} \right)$ be the processing of tfSKQ, where ${\mathcalligra{p}}$ is the query point, ${\mathcal{K}}$ is a query keyword set, $k$ is the number of expected query results, ${\mathbb{I}}$ is the RCL-tree index structure, and ${\mathcal{D}} = \left\{ {{\mathcalligra{d}}_{1} ,{\mathcalligra{d}}_{2} , \ldots ,{\mathcalligra{d}}_{k} } \right\}$ is the query results with the highest scores $\tau \left( {\mathcalligra{d}} \right)$, $\tau \left( {{\mathcalligra{d}}_{1} } \right) \ge \tau \left( {{\mathcalligra{d}}_{2} } \right) \ge \cdots \ge \tau \left( {{\mathcalligra{d}}_{k} } \right)$. Supported by ${\mathbb{I}}$, $Q_{f}$ firstly finds out R-tree nodes and corresponding concept lattices that meet the query conditions, ${\mathcalligra{p}}$, and ${\mathcal{K}}$, then retrieves and scores the spatial objects from concept lattices based on their frequency and spatial proximity, and finally returns k highest score spatial textual objects set ${\mathcal{D}}$.

The score of query result is defined by Eqs. (4)– (6).

4 $$freq\left( {{\mathcalligra{d}}_{i} } \right) = size\left( {Concept\left( {\mathcal{K}} \right).SubContect\left( {{\mathcalligra{d}}_{i} } \right).extent} \right)$$

5 $$dist\left( {{\mathcalligra{d}}_{i} } \right) = 1 - dist\left( {{\mathcalligra{p}},{\mathcalligra{d}}_{i} } \right)/\max \left( {dist} \right)$$

6 $$\tau \left( {{\mathcalligra{d}}_{i} } \right) = freq\left( {{\mathcalligra{d}}_{i} } \right) + dist\left( {{\mathcalligra{d}}_{i} } \right)$$

The score of frequency, $freq\left( {{\mathcalligra{d}}_{i} } \right)$, of ${\mathcalligra{d}}_{i}$, defined in Eq. (4), is the size of extents of the concept that includes the extent ${\mathcalligra{d}}_{i}$ and is the sub concept of the concept with the intent ${\mathcal{K}}$ in queried concept lattice. Since the concept in concept lattice presents the aggregation features (intent) of spatial objects (extent), spatial objects that meet query conditions ${\mathcal{K}}$ must be in the concept $Concept\left( {\mathcal{K}} \right)$, and their frequency is the number of spatial objects with the most typical feature. It can be considered that the most typical feature is the intent of the concept with the most objects. And low-level concept has less extent and more intent than high-level concept. Therefore, the most typical feature is the intent of the sub concept of $Concept\left( {\mathcal{K}} \right)$, and the frequency is the number of extents of the sub concept of $Concept\left( {\mathcal{K}} \right)$.

The score of the spatial proximity $dist\left( {{\mathcalligra{d}}_{i} } \right)$ defined in Eq. (5) is a normalized index with [0,1] value range and is inversely proportional to the Euclidean distance between the query point ${\mathcalligra{p}}$ and query result ${\mathcalligra{d}}_{i}$. Then the score of query results $\tau \left( {{\mathcalligra{d}}_{i} } \right)$ defined in Eq. (6) is the sum of $freq\left( {{\mathcalligra{d}}_{i} } \right)$ and $dist\left( {{\mathcalligra{d}}_{i} } \right)$.

Note that the frequency score $freq\left( {{\mathcalligra{d}}_{i} } \right)$ is an integer greater than 0, and the distance score $dist\left( {{\mathcalligra{d}}_{i} } \right)$ is a decimal from 0 to 1. In this way, the frequency has the higher priority than the distance. When the frequencies of spatial objects are the same, the distance score will be considered, and thus, the most frequent items are retrieved first and then sorted by the spatial proximity.

The implementation algorithm of $Q_{f}$ is shown in Algorithm 2 and its inputs are a query point ${\mathcalligra{p}}$, a query keywords set ${\mathcal{K}}$, the number of expected results $k$, and the RCL-tree ${\mathbb{I}}$. Its output is a query results set ${\mathcal{D}}$ with k highest scoring objects. The process of $node\_query$, i.e. Algorithm 3, is executed first and a tree node set ${\mathcal{N}}$ that include ${\mathcalligra{p}}$ and link to concept lattice are retrieved from ${\mathbb{R}}$. Then, the concept lattice structure set ${\mathcal{L}}$ linked to ${\mathcal{N}}$ is obtained by the $lattice\_query$ process, i.e. Algorithm 4. In the $frequent\_score$ process, i.e. Algorithm 5, spatial textual objects are extracted from ${\mathcal{L}}$ and scored with Eq. (4). According to their score, the set ${\mathcal{D}}$ of top k frequent spatial textual objects are retrieved, and tfSKQ is answered.
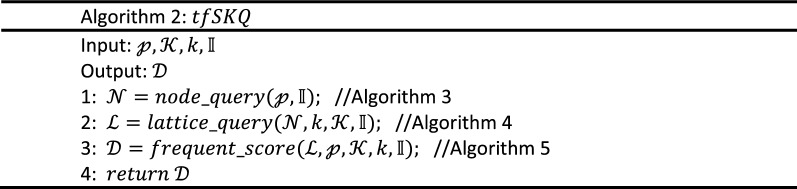


Because of the connectivity between R-tree node and concept lattice structure, we must first find out tree nodes that meet spatial query condition ${\mathcalligra{p}}$ and link with concept lattices. In Algorithm 3, a rough spatial query is executed, and several tree nodes linked to concept lattice with minimum R-tree $level$ are retrieved. Its inputs are the query point ${\mathcalligra{p}}$, the proposed RCL-tree index structure ${\mathbb{I}}$. And its output is a R-tree node set ${\mathcal{N}}$.

Because these $mbr$ s of R-tree nodes are allowed to overlap, there may be multiple nodes meeting the spatial query condition ${\mathcalligra{p}}$. We employ stack structure to achieve top-down traverse of nodes in ${\mathbb{I}}.{\mathbb{R}}$, and satisfied nodes are filtered by two criteria. Criteria 1 (line 5): the spatial proximity criteria, which is employed to retrieve the tree nodes in ${\mathbb{I}}.{\mathbb{R}}$ that include the query point ${\mathcalligra{p}}$. Criteria 2 (line 6): the minimum concept lattice criteria, on the basis of Criteria 1, which is employed to search the minimum level tree nodes linked with concept lattice structure, in other word, the selected tree nodes are the nodes that include the query point ${\mathcalligra{p}}$ and link to a concept lattice with the minimum tree $level$. Then the selected nodes and their sibling nodes are inserted into the result set ${\mathcal{N}}$. Theoretically, the result quality of Algorithm 3, i.e., the query quality of spatial proximity, is related to the parameter $\delta$ which determines the level of tree nodes linking concept lattice, and then affects the degree of spatial proximity. To fully mine the textual keyword features in subsequent algorithms, a moderate number of spatial objects need to be retrieved and the value range of $\delta$ need to be tuned and optimized (some results of two real datasets are shown in “RCL-tree evaluation” section).
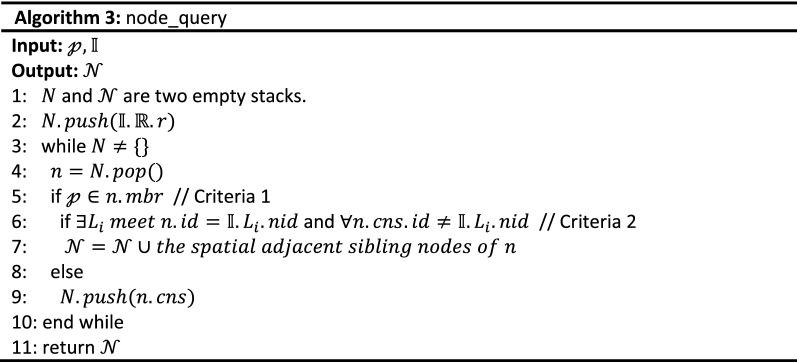


The relationship between concept lattice and R-tree node in RCL-tree is not one-to-one, only partial R-tree nodes (the size of their formal context is within $\left[ {\delta_{min} ,\delta_{max} } \right]$, see in “Methodology” section) link to concept lattices. Therefore, Algorithm 4 is to find suitable concept lattices for the R-tree node set ${\mathcal{N}}$ returned from Algorithm 3 and containing $k$ results meeting ${\mathcal{K}}$. The inputs of Algorithm 4 are the R-tree node set ${\mathcal{N}}$, a query keywords set ${\mathcal{K}}$, the number of expected results $k$, and RCL-tree ${\mathbb{I}}$. Its output is a set of concept lattice ${\mathcal{L}}$.

For each node $n$ in ${\mathcal{N}}$, add the concept lattice linked with $n$ to ${\mathcal{L}}$ (line 3 to 4) and use $ki$ represent the extent number of satisfied concepts that meet ${\mathcal{K}}$ in a concept lattice, and use $kn$ represent the total number of the extents in all concept lattice (line 2 to 6). If $kn$ doesn’t meet the number of expected results $k$, i.e. $kn < k$, reverse ${\mathcal{L}}$ and search $\ell p$ that contains more eligible extends, i.e. $\ell .nid = n.id$ and ${\mathcalligra{l}}p.nid = n.pn.id$ (line 8 to 16), until $k$ results satisfying ${\mathcal{K}}$ are found out. At last, the concept lattice set ${\mathcal{L}}$ containing $k$ results satisfying ${\mathcal{K}}$ is output.
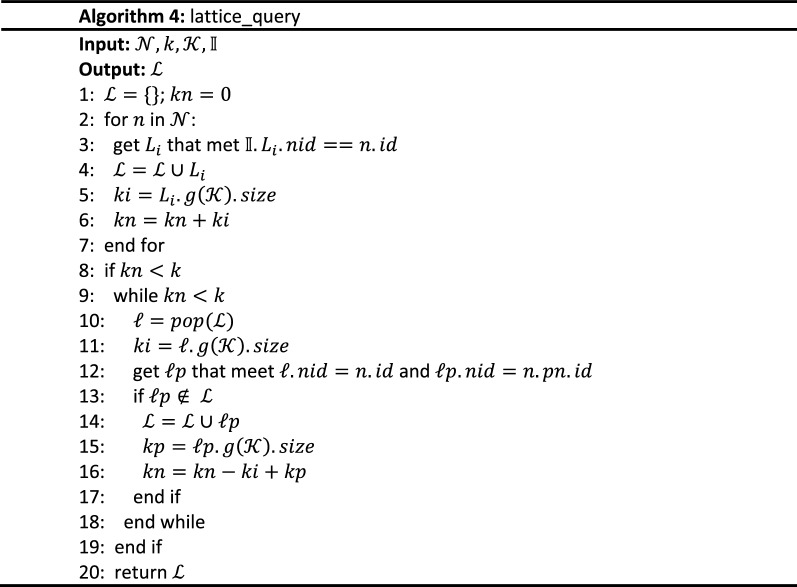


The inputs of Algorithm 5 are a concept lattice set ${\mathcal{L}}$ from Algorithm 4, a query point ${\mathcalligra{p}}$, a query keywords set ${\mathcal{K}}$, the number of expected results $k$, the RCL-tree ${\mathbb{I}}$. Its output is the top k frequent spatial textual objects, i.e. the query results of tfSKQ.

Based on Eq. (6), Algorithm 5 traverses each concept $C$ of concept lattice in ${\mathcal{L}}$ to search satisfying concepts, then extracts spatial textual objects to ${\mathcal{D}^{\prime}}$ and measures their scores (line 2 to 12). Next, sort these objects by their scores and take the first k objects (line 13 to 14). Finally, measures the spatial proximity score of them and sort ${\mathcal{D}^{\prime}}$ to ${\mathcal{D}}$ (line 15 to 20), outputs ${\mathcal{D}}$ (line 21), finishes tfSKQ.
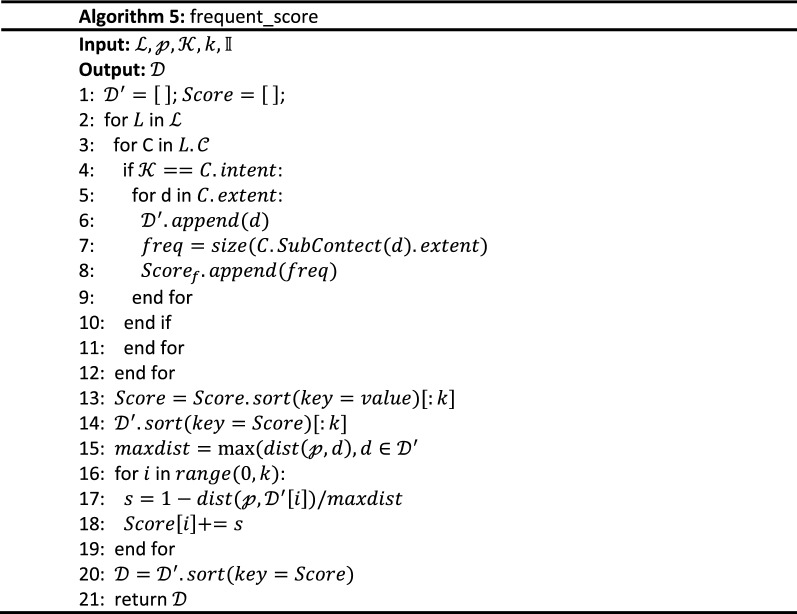


To the aspect of time complexity, the tfSKQ algorithm has the logarithmic retrieval efficiency because the tree structure and the lattice structure in RCL-tree are such retrieval efficiency^15,16^. To retrieve the tree nodes around the target point, Algorithm 3 traverses the tree structure of RCL-tree and checks the sibling nodes of the nodes containing the query point and returns the tree node set ${\mathcal{N}}$ with $O\left( {nlogn} \right)$. Algorithm 4 traverses ${\mathcal{N}}$ to get the corresponding concept lattice set ${\mathcal{L}}$ with $O\left( n \right)$. Algorithm 5 extracts the most frequent objects from each concept lattice with $O\left( {nlogn} \right)$, scores them based on Eqs. (4)–(6) and return the k most frequent spatial objects with $O\left( {n^{2} } \right)$.

## Data and experiment

To evaluate the performance of the proposed RCL-tree and tfSKQ algorithm, we conduct a series of comparative experiments with some existing methods using the actual STBD set. Later, after processing, we evaluated their effectiveness and efficiency, accordingly using tables and figures as presented below.

### Data preparation and preprocessing

This paper employs two spatial textual datasets to evaluate the proposed RCL-tree and tfSKQ algorithm. One is a real business dataset from “Yelp Open Dataset” (yelp.com/dataset), named “Yelp”, which contains about 192,609 businesses, including 8 fields such as “*business_id*”, “*latitude*”, “*longitude*”, “*starts*”, “*review_count*”, “*is_open*”, “*attributes*”, “*categories*”, etc. The other is a POI dataset from AutoNavi (www.amap.com), named “Amap”, which contains 483,991 business POIs in Shanghai, China. Because the concept lattice structure in the RCL-tree accepts the binary fields only, the above two raw dataset need to be preprocessed as the binary formal context with multi textual keywords.

For Yelp, we select some important fields from the business dataset and design a binary formal context with 41 columns divided into five categories, as shown in Table 1. The first 26 columns are from the “*categories*” field and cover the business dataset completely. In other words, every record of the business dataset satisfies one or more of them. Columns 27–29 from “*review_count*” discretize the number of reviews into three grades: Rc_low, Rc_middle, Rc_high based on the tri-sectional quantiles of “*review_count*”. Columns 30–32 discretize the “*stars*” into three grades: S_low, S_middle, S_high in [0,2], [2.5,3.5] [4, 5]. Columns 33 is from “*is_open*” and represents the operation status of object. Columns 34–41 selected from “*attributes*” include 8 common features of business that covered about 85% of total data records with one or more than 1 value, while other 15% records are all of 0 value in these 8 columns.

**Table 1:** The column structure of binary formal context from the Yelp business dataset.

Columns	Number	Column Name	Raw field
1–26	26	Beauty & Spas, Education, Health & Medical, Automotive, Bars, Mass Media, Event Planning & Services, Financial Services, Local Services, Local Flavor, Gyms, Parks, Home Services, Fitness & Instruction, Pets, Shopping, Religious Organizations, Active Life, Landscape Architects, Public Services & Government, Restaurants, Hotels & Travel, Professional Services, Arts & Entertainment, Nightlife, Food	*Categories*
27–29	3	Rc_low, Rc_middle, Rc_high	*review_count*
30–32	3	S_low, S_middle, S_high	*stars*
33	1	Is_open	*is_open*
34–41	8	Alcohol, DogsAllowed, GoodForDancing, HasTV, Music, Open24Hours, Smoking, WIFI	*attributes*

Then, ${\mathbb{D}}_{Yelp} = \{ d_{i} |1 \le i \le 192,609\}$, the textual keywords set $K = \{ \left\langle {k_{1} ,k_{2} , \ldots ,k_{j} , \ldots ,k_{41} } \right\rangle {|} k_{j} \in \left\{ {0,1} \right\},1 \le j \le 41\}$, and the average keywords coverage is about 11% that means each spatial object has about 4.5 keywords on average.

For Amap, except for location and category information, it has no keywords suitable for the binary formal context. To ensure the comparability of experimental results, we also want to design 41 simulation textual keywords similar to Yelp to modify Amap. In addition, to present the effect of data complexity on retrieval performance, we set the average keywords coverage to 17%, about 7 keywords of per spatial object, to construct the Amap dataset. Then, ${\mathbb{D}}_{Amap} = \{ d_{i} |1 \le i \le 483,991\}$, the textual keywords set $K = \{ \left\langle {k_{1} ,k_{2} , \ldots ,k_{j} , \ldots ,k_{41} } \right\rangle {|} k_{j} \in \left\{ {0,1} \right\},1 \le j \le 41\}$, and the average keywords coverage of Amap is 17%, about 7 keywords per spatial object.

All of experiments are performed on Python 3.7 with a computer equipped with Intel i5, 3.0 GHz CPU, 24 GB RAM, and 64bit Windows 10 operation system.

### RCL-tree evaluation

To initialize the RCL-tree index structure, Algorithm 1 (see in “Initialization algorithm” section) need to be conducted, and two thresholds, $\theta$ and $\delta$, need to be determined in advance. $\theta$ is the range of R-tree node entries, and $\delta$ is the range of data volume of R-tree node linked to concept lattice. In general, $\theta$ is designed to have a similar number of entries for nodes to balance the retrieval time. In addition, for RCL-tree, few node entries make simple node structure and is helpful to link to concept lattice efficiently. Therefore, let $\theta_{Yelp} = \left[ {2,4} \right]$ be the range of R-tree node entries in Yelp. The R-tree structure of RCL-tree in Yelp can be built, and 291,678 tree nodes are generated, including $192,609$ leaf nodes, ${\mathbb{R}}_{{{\varvec{Yelp}}}} = \{ n_{1} ,\left[ {2,4} \right],{ }\left\langle {n_{1} , n_{2} , \ldots ,n_{i} , \ldots ,n_{291,678} } \right\rangle |1 \le i \le 291,678\}$, ${\mathbb{R}}_{{{\varvec{Yelp}}}} .root = n_{1}$.

**Figure 4:**
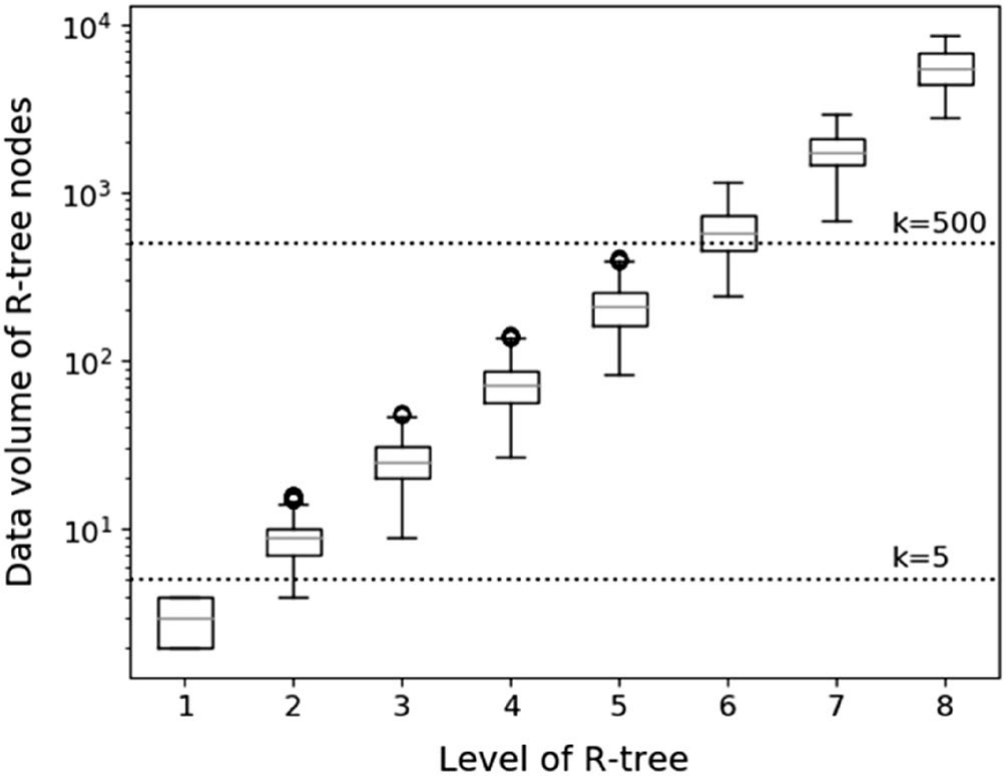
The statistical features of data volume of R-tree nodes in ${\mathbb{R}}_{Yelp}$.

$\delta$ is an important factor to determine how many concept lattices should be built. Since tfSKQ is to retrieve the *k* objects by traversing concept lattices, we expect that the *k* query results can be obtained by traversing as few concept lattice structures as possible, in other word, we expect the *k* and the data volume of concept lattice have a similar value range. To achieve it, we explore the detailed statistical features of R-tree nodes in ${\mathbb{R}}_{{{\varvec{Yelp}}}}$ in Yelp, and the results are shown in Fig. 4 and Table 2. In Fig. 4, the box diagrams of data volume of R-tree nodes in level 1–8 (the maximum level ${\mathbb{R}}$ is 11) of are drawn based on the level of R-tree nodes. And the nodes of level 2–5 are in the range of [5, 500] of *k*, which is a widely recognized query range and often used in a variety of related literatures. We can create concept lattice structures linked with these R-tree nodes in level 2–5 one by one to meet the efficient tfSKQ. However, as you can see from Table 2, the number of nodes in level 2, 22,149, is too large to the initialization of RCL-tree, and the minimum value of nodes in level 2 is 4, which means that a considerable number of nodes in level 2 do not meet the query number *k*. Therefore, for the yelp business datasets, we employ these level 3–5 R-tree nodes to build concept lattices one by one and set $\delta_{Yelp} = \left[ {9,413} \right]$, covering all 11,142 tree nodes in levels 3–5. Then, 11,142 concept lattices are built, and ${\mathbb{L}}_{Yelp} = \{ L_{1} ,L_{2} , \ldots ,L_{i} , \ldots ,L_{11142} {|}1 \le i \le 11,142, L_{i} .F.size \in \left[ {9,413} \right]$}, the RCL-tree of Yelp is initialized, ${\mathbb{I}}_{Yelp} = \left\langle {{\mathbb{R}}_{Yelp} ,\user2{ }{\mathbb{L}}_{Yelp} } \right\rangle$.

**Table 2:** The statistics of data volume of node by tree level.

Data volume of nodes	Tree level										
	1	2	3	4	5	6	7	8	9	10	11
Count	65,293	22,149	7591	2636	915	322	110	35	12	4	1
Mean	3.0	8.7	25.3	73.1	210.5	598.2	1751.0	5503.1	16,050.8	48,152.3	192,609
std	0.8	2.5	7.5	22.4	65.1	192.3	478.5	1595.2	5421.9	5985.1	
Min	2	4	9	27	83	245	685	2778	7554	43,728	192,609
25%	2	7	20	56	161	452	1450.3	4397.5	13,259	44,190.8	192,609
50%	3	9	25	72	209	583	1749.5	5418	15,731.5	46,084	192,609
75%	4	10	31	88	253	740	2079	6774.5	18,845	50,045.5	192,609
Max	4	16	49	145	413	1146	2925	8547	27,846	56,713	192,609

We also conduct similar experiments on Amap, based on these same principles, the RCL-tree of Amap is created. Specifically, $\theta_{Amap} = \left[ {2,4} \right]$, 732,342 R-tree nodes are generated, and ${\mathbb{R}}_{Amap} = \{ n_{1} ,\left[ {2,4} \right],{ }\left\langle {n_{1} , n_{2} , \ldots ,n_{i} , \ldots ,n_{732,342} } \right\rangle |1 \le i \le 732,342\}$. Concept lattices are created on 27,930 R-tree nodes in level 3–5, $\delta_{Amap} = \left[ {8,458} \right]$, and ${\mathbb{L}}_{Amap} = \{ L_{1} ,L_{2} , \ldots ,L_{i} , \ldots ,L_{27930} {|}1 \le i \le 27930, L_{i} .F.size \in \left[ {8,458} \right]$, ${\mathbb{I}}_{Amap} = \left\langle {{\mathbb{R}}_{Amap} ,\user2{ }{\mathbb{L}}_{Amap} } \right\rangle$.

Table 3 shows the details of the initialized RCL-tree on ${\mathbb{D}}_{Yelp}$ and ${\mathbb{D}}_{Amap}$. Only 3.8% R-tree nodes need to link to concept lattice, thus saving storage space and improving initiation efficiency. In addition, the number of concepts in concept lattice is greater than the number of objects, which represents the complexity of textual keywords. The more the complexity in the textual keywords of objects, the more the concepts in concept lattices.

**Table 3:** The details of RCL-tree index structure.

Description	Yelp	Amap
Dataset size (MB)	131	209
Total of spatial textual objects	192,609	483,991
The size of ${\mathbb{I}}$ (MB)	345	3726
The size of ${\mathbb{I}}.{\mathbb{R}}$ (MB)	41	130
The size of ${\mathbb{I}}.{\mathbb{L}}$ (MB)	304	3596
The number of tree nodes in ${\mathbb{I}}.{\mathbb{R}}$	291,678	732,342
The number of concept lattices in ${\mathbb{I}}.{\mathbb{L}}$	11,142	27,930
The percentage of R-tree nodes linked with concept lattices	3.8%	3.8%
Average number of concepts in concept lattice	75	261
Average number of objects in concept lattice	52	52

**Figure 5:**
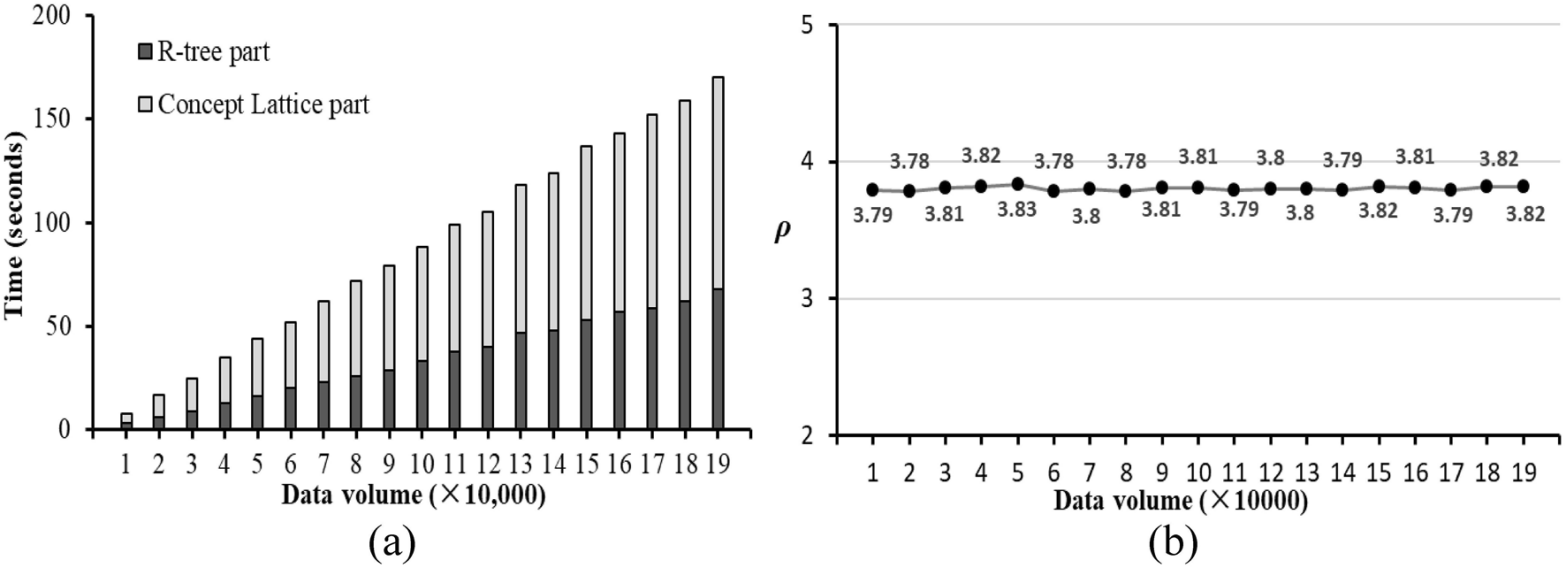
The influences of data volume on ${\mathbb{L}}_{Yelp}$. (**a**) The initialization time of ${\mathbb{L}}_{Yelp}$ with different data volume, (**b**) The size ratio of $\rho_{Yelp}$ with different data volumes.

To evaluate the efficiency of RCL-tree initialization process (Algorithm 1), the influences of data volume on ${\mathbb{D}}_{Yelp}$ are demonstrated by Fig. 5. As shown in Fig. 5a, dark colour rectangles represent the initialization time of ${\mathbb{R}}_{Yelp}$ in ${\mathbb{I}}_{Yelp}$, and light colour rectangles represent the initialization time of ${\mathbb{L}}_{Yelp}$, and the initialization time of ${\mathbb{I}}_{Yelp}$ is the sum of them. Obviously, ${\mathbb{R}}_{Yelp}$ time is always less than ${\mathbb{L}}_{Yelp}$ time. And with the increase of data volume, the initialization time of ${\mathbb{I}}_{Yelp}$ increases linearly. For ${\mathbb{D}}_{Yelp}$, included 192,609 spatial textual objects, the time of ${\mathbb{I}}_{Yelp}$, ${\mathbb{R}}_{Yelp}$, and ${\mathbb{L}}_{Yelp}$ is about 175 s, 69 s, and 106 s.

In addition, we analyse the quantitative relationship between ${\mathbb{L}}_{Yelp}$ and ${\mathbb{R}}_{Yelp}$. Let $\rho_{Yelp} = 100 \times {\mathbb{L}}_{Yelp} .size/{\mathbb{R}}_{Yelp} .{\text{size}}$ be the ratio of the number of concept lattices in ${\mathbb{L}}_{Yelp}$ to the number of nodes in ${\mathbb{R}}_{Yelp}$. Figure 5b shows the trends of $\rho_{Yelp}$ with different data volumes. As you can see, $\rho_{Yelp}$ always fluctuates around 3.8. Therefore, we can think that the setting of $\delta_{Yelp}$ is reasonable and adequate. Similar conclusions, $\rho_{Amap} \approx 3.8$, can also be obtained on Amap and will not be repeated here, since the same setting of $\delta_{Yelp}$ and $\delta_{Amap}$ that they all build concept lattices at level 3 to 5.

### The evaluation and comparison of tfSKQ

Based on the RCL-tree, the proposed tfSKQ algorithm takes spatial point ${\mathcalligra{p}}$ and textual keywords ${\mathcal{K}}$ as the query conditions to retrieve the $k$ most frequent and nearest items on ${\mathbb{D}}_{Yelp}$ and ${\mathbb{D}}_{Amap}$. Different with the common top-k spatial keyword query (TkSKQ), tfSKQ can not only express spatial proximity but also reveal the textual keyword aggregation features of spatial objects to present the frequent items and its frequency.

To evaluate the performance of the proposed tfSKQ algorithm shown in Algorithm 2–5, a similar algorithm proposed by Ahmed et al*.*^10^ is employed. Ahmed proposes a hybrid index structure with a R-tree and some *top-k sorted term lists* (STLs), and develops algorithms to efficiently answer the *top-k frequent spatiotemporal terms* (kFST) query. Similar with IR-tree^1^, STLs index structure employ inverted structure to store *sorted keyword lists* in tree nodes and leaf nodes of the R-tree structure, but the difference is that STLs maintain the frequency of each keyword in nodes. To make the STLs index and RCL-tree comparable, we use the parameter $\delta$ of RCL-tree to limit tree nodes linked to *sorted term lists* in STLs index, that is to say, in STLs index, only the level 3 to 5 R-tree nodes connect with *sorted term lists*. We call this variant of the SLTs index as **δSTLs**. Note that, since δSTLs only stores single keyword’s frequency in STLs, it can only answer the frequency with the 0 textual keyword, i.e. ${\mathcal{K}} = \{ \}$, and cannot analyse the frequency of complex multiple keywords combinations.

We also compare tfSKQ with two classical frequent items algorithms Apriori^17^ and FP-Growth^18^. Apriori algorithm employs the support degree as the criterion of judging frequent items to find the largest multiple frequent items. FP-Growth algorithm constructs a *frequent pattern tree* (FP-tree), maps data to the tree, and finds all frequent FP-tree items. Based on them, we develop two baseline index schemas to compare with RCL-tree and tfSKQ algorithm.

One is the combination of a R-tree structure and some frequent item tables generated by Apriori algorithm, named **A-frequent**. It employs a R-tree structure to index the spatial information and employs some frequent item tables generated by Apriori algorithm to store the frequent items of the textual keyword information of each R-tree node. Each record in the frequent item table includes two columns $\left\langle {frequent item, frequency} \right\rangle$, i.e. the frequent item and its frequency. A-frequent method can retrieve the k most frequent items to answer tfSKQ by the query conditions and the minimum support degree parameter. The second is the hybrid of R-tree and FP-tree, named **F-frequent**. It employs a R-tree structure and some FP-tree structures to index spatial information and textual keywords of each R-tree node respectively. The tfSKQ can be solved by the given query conditions and the minimum support parameter.

Like RCL-tree, A-frequent and F-frequent are both limited by $\delta$, i.e. frequent item tables in A-frequent and FP-tree structures in F-frequent are both built in level 3 to5 R-tree nodes. In addition, in A-frequent and F-frequent methods, the minimum support degree for querying frequent items is set to 0.1%.

Then, the RCL-tree is compared with the above three methods, δSTLs, A-frequent, and F-frequent, in Yelp and Amap dataset, and the results are as follows.

**Figure 6:**
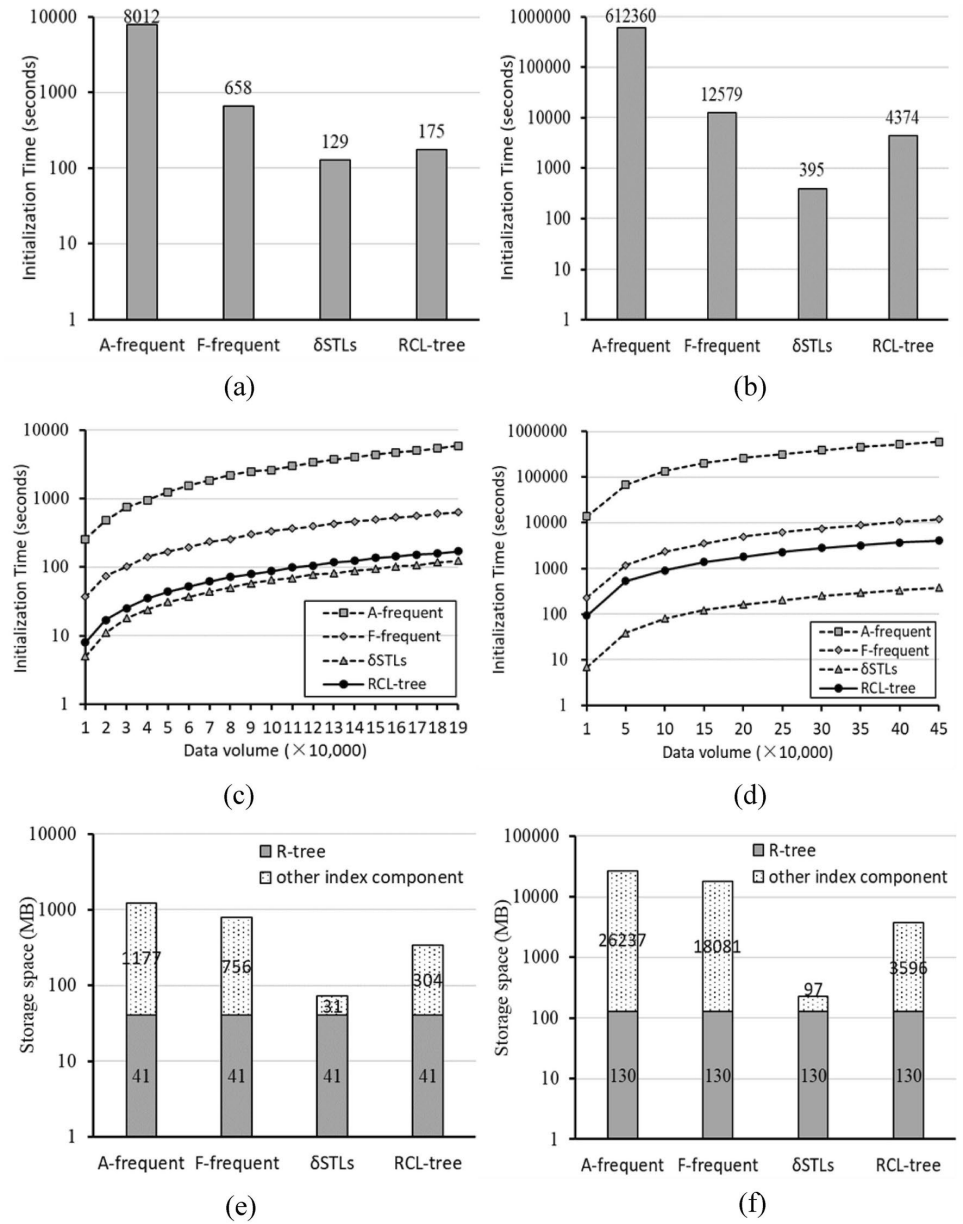
The comparison of initialization. (**a**) ${\mathbb{I}}_{Yelp}$ initialization time, (**b**) ${\mathbb{I}}_{Amap}$ initialization time, (**c**) Effect of varying data volume for ${\mathbb{I}}_{Yelp}$, (**d**) Effect of varying data volume for ${\mathbb{I}}_{Amap}$, (**e**) the comparison of storage space in Yelp, (**f**) the comparison of storage space in Amap.

Figure 6 shows the comparisons of initialization time. In Fig. 6a,b, since δSTLs only stores single keyword’s frequency, it has the shortest total initialization time 129 s in Yelp and 395 s in Amap, while the other three methods need longer time to maintain all frequent information including the frequency information of single keyword and multiple keywords. Except for δSTLs, others increase dramatically from Yelp to Amap, A-frequent increases by about 76 times, F-frequent 19 times, and RCL-tree 25 times, while the increase of data volume is about 2,5 times. It means that the maintenance cost of frequent items is affected by data volume, and is more related to the complexity of data itself. These differences are also shown in Fig. 6c,d, with the increase of data volume, the initialization time gaps between them remain unchanged. In addition, since A-frequent employ table structure to maintain frequent information, there are many table-based traversal operations and a large number of data insertions and update in the initialization of A-frequent method, A-frequent always has the much longer initialization time in ${\mathbb{I}}_{Yelp}$ and ${\mathbb{I}}_{Amap}$ than others. Compared with A-frequent, F-frequent uses tree structure to do it and RCL-tree uses lattice structure. Among the three methods that store multiple keywords frequent information, as shown in Fig. 6a–d, RCL-tree always has the shortest initialization time.

Comparative results of storage space are given in Fig. 6e,f. In Yelp, with a R-tree structure 41 MB and some frequent item tables 1177 MB, A-frequent has the maximum storage space, 1218 MB. δSTLs has the minimum storage space of 72 MB with a R-tree structure 41 MB and some STLs 31 MB, because only the frequent information about single keyword is stored in it. And F-frequent, RCL-tree are 797 MB, 345 MB with FP-tree set 756 MB and concept lattices 304 MB respectively. Similar differences of them are also show in Fig. 6f, with the same R-tree 130 MB, the other component of A-frequent has the maximum 26237 MB, followed by F-frequent 18081 MB and RCL-tree 3596 MB, and δSTLs 97 MB at least. It indicates that these four index structures have the same R-tree component, and when multi keyword frequent information is stored, the concept lattices component in RCL-tree is the most compact and efficient storage structure than FP-tree of F-frequent, and the frequent item tables of A-frequent.

Next, the comparison of retrieval time of tfSKQ are conducted by three aspects: data volume, the number of query results, and the number of query keywords, are as below. Note that, because of the uneven distribution of spatial objects, random query points of tfSKQ often bring different query results, which gives difficult to objectively present the algorithm performance. To avoid it, the results of each query experiments are the average of 100 experiments under the same query conditions.

**Figure 7:**
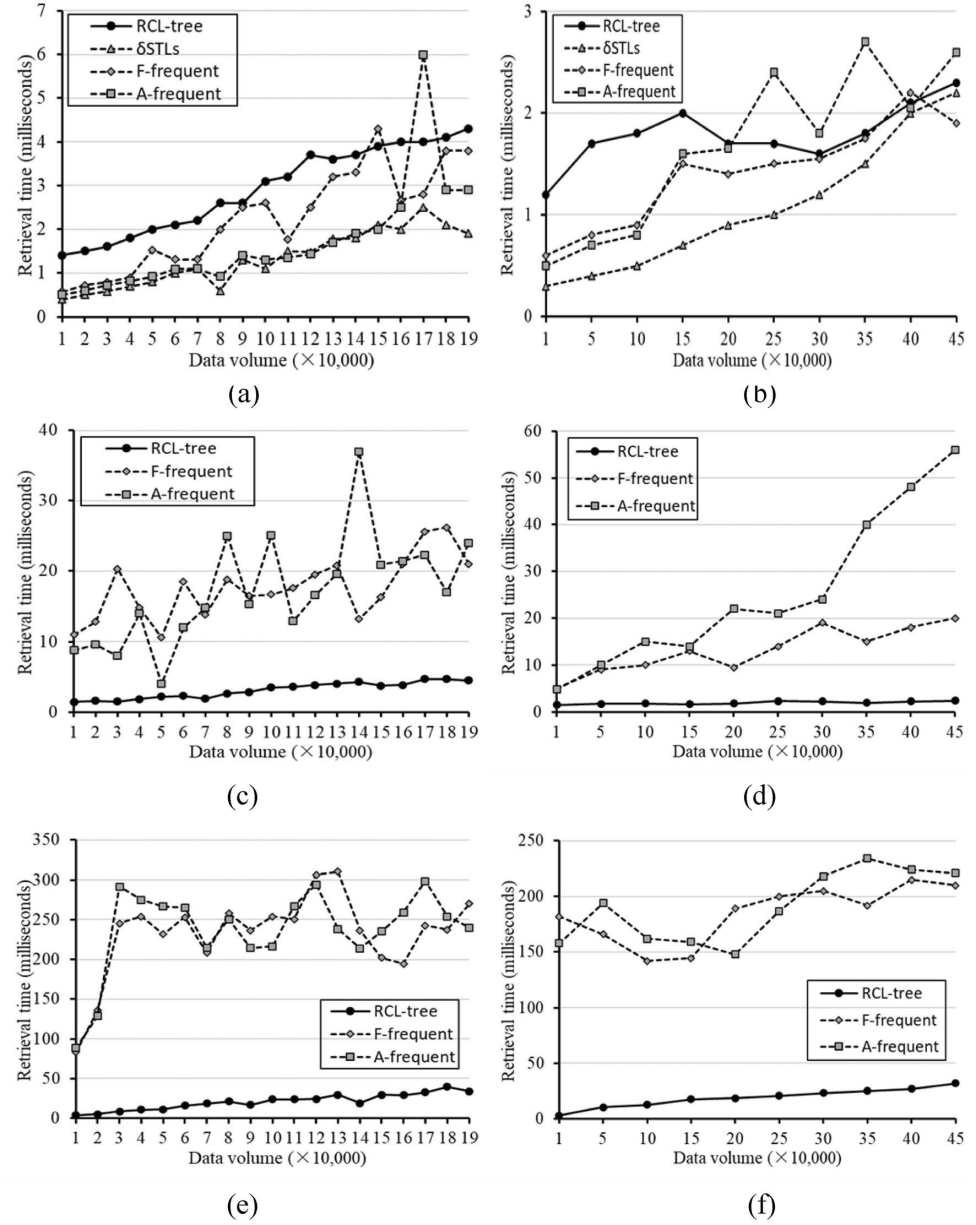
Effect of data volume and the number of query keywords on retrieval time. (**a**) ${\mathbb{I}}_{Yelp}$ with ${\mathcal{K}}.size = 0$, (**b**) ${\mathbb{I}}_{Amap}$ with ${\mathcal{K}}.size = 0$, (**c**) ${\mathbb{I}}_{Yelp}$ with ${\mathcal{K}}.size = 1$, (**d**) ${\mathbb{I}}_{Amap}$ with ${\mathcal{K}}.size = 1$, (**e**) ${\mathbb{I}}_{Yelp}$ with ${\mathcal{K}}.size = 2$, (**f**) ${\mathbb{I}}_{Amap}$ with ${\mathcal{K}}.size = 2$.

Firstly, the effects of data volume on retrieval time are given in Fig. 7. Under the different number of query keywords and *k* = 10, the tfSKQ results of these four methods are significantly different. Because δSTLs can only be applied to tfSKQ with empty keyword query condition, i.e. ${\mathcal{K}}.size = 0$ or ${\mathcal{K}} = \{ \}$, δSTLs only participates in the comparative experiments of ${\mathcal{K}}.size = 0$. Shown in Fig. 7a,b, STLs has the best performance than others, RCL-tree has the worse retrieval time in some cases, and the retrieval time of A-frequent and F-frequent dose not grow steadily with the increase of data volume. In Fig. 7c–f, the query keyword set ${\mathcal{K}}$ is not an empty set, the results are reversed, the retrieval time of RCL-tree is significantly better than that of A-frequent and F-frequent in both of ${\mathbb{I}}_{Yelp}$ and ${\mathbb{I}}_{Amap}$. And these gaps are more pronounced in ${\mathbb{I}}_{Amap}$. That is because the frequent items stored by δSTLs, A-frequent, and F-frequent are ordered and the frequency of single keyword is easier to retrieve, while the frequent items stored by RCL-tree are generalized as concepts, and the frequency of keyword need to be deduced from concept lattice. In addition, it can be seen that the retrieval time of A-frequent and F-frequent are unstable in all three cases, and they grow leaps and bounds with the increase of data volume, while the retrieval time of RCL-tree always increases linearly with the increase of data volume. It indicates that RCL-tree has better robustness and adaptability than other methods in complex tfSKQ.

**Figure 8:**
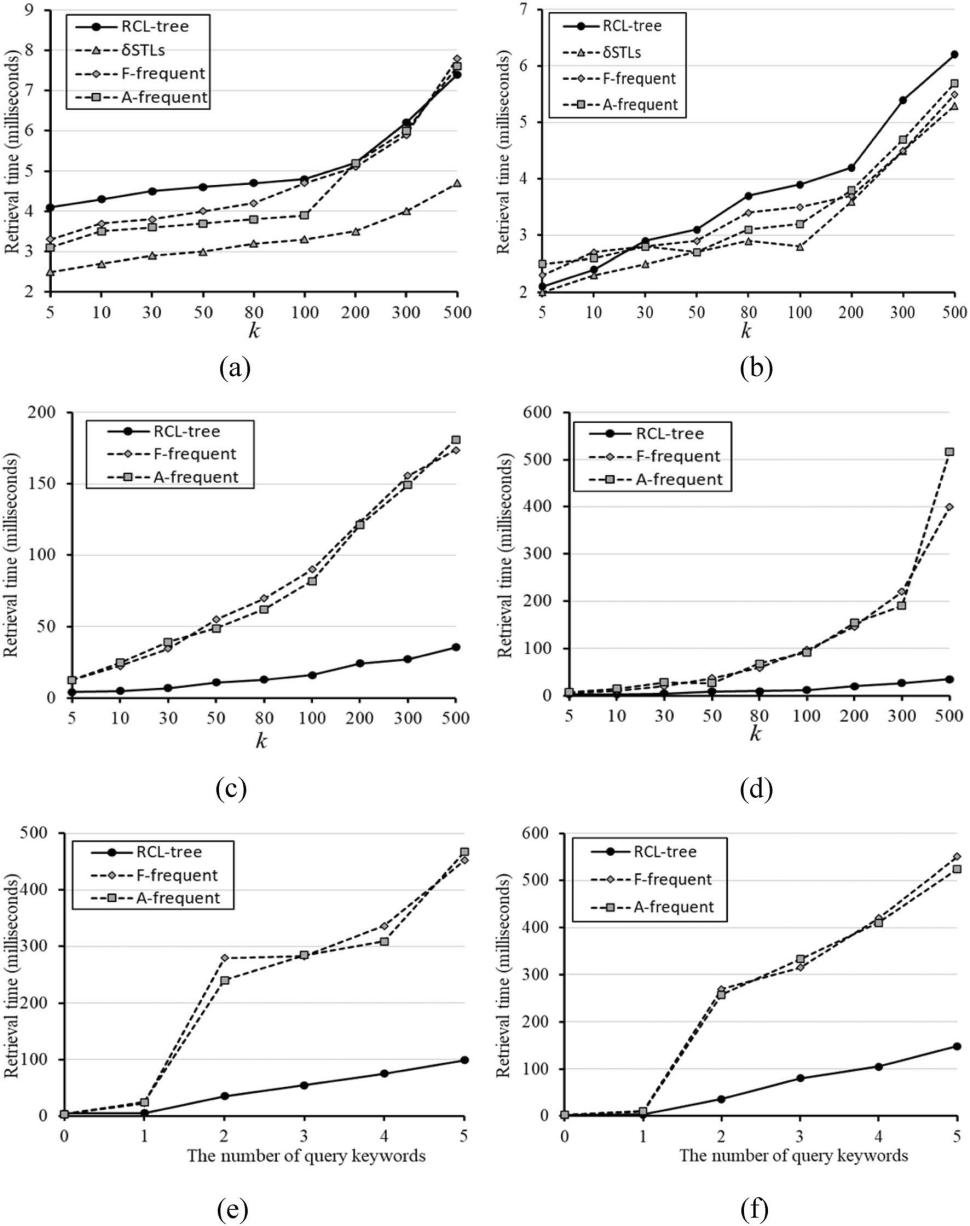
Effect of *k* and the number of query keywords on retrieval time. (**a**) ${\mathbb{I}}_{Yelp}$ with ${\mathcal{K}}.size = 0$, (**b**) ${\mathbb{I}}_{Amap}$ with ${\mathcal{K}}.size = 0$, (**c**) ${\mathbb{I}}_{Yelp}$ with ${\mathcal{K}}.size = 1$, (**d**) ${\mathbb{I}}_{Amap}$ with ${\mathcal{K}}.size = 1$, (**e**) ${\mathbb{I}}_{Yelp}$ with $k = 10$, (**f**) ${\mathbb{I}}_{Amap}$ with $k = 10$.

Figure 8 shows the effect of *k* and the number of query keywords on retrieval time with the full data set. In Fig. 8a–d, we still employ the number of query keywords as a factor to observe the performance of these four methods. Figure 8a,b show the effect of *k* with ${\mathcal{K}}.size = 0$ in ${\mathbb{I}}_{Yelp}$ and ${\mathbb{I}}_{Amap}$. We can see that δSTLs is still the best method, and RCL-tree is the worst one in most cases. This situation is changed when ${\mathcal{K}}.size = 1$. As shown in Fig. 8c,d, A-frequent and F-frequent have the same trends with the increase of *k*, the performance of RCL-tree is great better than that of A-frequent and F-frequent, and the gap between them grows with the increase of *k*. When *k* = 500, the retrieval time of ${\mathbb{I}}_{Yelp}$ is 35.6 ms, which is about 1/5 of A-frequent 173.7 ms and F-frequent 181.1 ms, and ${\mathbb{I}}_{Amap}$ is 35.0 ms, which is about 1/15 of A-frequent 517.0 ms and 1/12 of F-frequent 400.0 ms.

Obviously, RCL-tree has more advantages than other methods when ${\mathcal{K}}$ is not an empty set. The detailed analysis about the effect of ${\mathcal{K}}$ on retrieval time with *k* = 10 and the full data set are shown in Fig. 8e,f. We can see that as the number of query keywords increases, the process of tfSKQ becomes more complex, and the advantages of RCL-tree is more obvious. When the number of query keywords is 5, the retrieval time of ${\mathbb{I}}_{Yelp}$ is 98.8 ms, which is about 1/5 of F-frequent 452.0 ms and A-frequent 466.9 ms. Similarly, ${\mathbb{I}}_{Amap}$ is 148 ms, and F-frequent 550.0 ms, A-frequent 524.0 ms.

In this section, we employ two datasets, Yelp and Amap, to compare the performance of RCL-tree with other three methods, δSTLs, A-frequent, and F-frequent, in initialization and tfSKQ. Although δSTLs performs well in keyword free query, it cannot directly achieve tfSKQ of multi keyword query due to its own structure. There is no doubt that in the case of multi keyword query, RCL-tree has the best efficiency in initialization and tfSKQ, its retrieval performance is at least 5 times of A-frequent and F-frequent, and its storage occupy is at least 2/5 of F-frequent and 1/4 of A-frequent. It is worth mentioning that, on two dataset Yelp and Amap, the RCL-tree has the stable performance and its retrieval efficiency is always better than other baseline methods.

## Conclusion

The complexity of textual keywords of STBD and their existing table-based index schemas make obstacles to efficient the tfSKQ, especially in the case of multi query keywords. This paper employs concept lattice structure to solve it for the first time. A novel hybrid index structure RCL-tree composed of R-tree and concept lattices and a tfSKQ algorithm are proposed to achieve efficient tfSKQ for STBD. The results of empirical researches demonstrate that RCL-tree outperforms some existing methods in terms of initialization and frequent items retrieval in the case of multi query keywords. The proposed solution for tfSKQ aims at not only filling the gap in the spatial frequent multi keywords query, but also promoting the application of spatial textual big data indexing.

Future research will focus on the following three directions. Firstly, to examine the applicability of tfSKQ with other more STBD sets. Secondly, to explore the scalability of the RCL-tree and develop a multi granularity index structure to support spatial–temporal frequent items. Lastly is to investigate and examine fundamental structures of the RCL-tree that can be revisited to explore the possibility of improving its performance.

## Data Availability

The data and code that support the findings of this study are available in “figshare.com” with the identifier: https://doi.org/10.6084/m9.figshare.15052236.

## References

[1] Cong G, Jensen CS, Wu D (2009). Efficient retrieval of the top-k most relevant spatial web objects. Proc. VLDB Endow..

[2] Cary A, Wolfson O, Rishe N, Gertz M, Ludäscher B (2010). Efficient and scalable method for processing top-k spatial boolean queries. Scientific and Statistical Database Management.

[3] Christoforaki, M., He, J., Dimopoulos, C., Markowetz, A. & Suel, T. Text vs. space: Efficient geo-search query processing. In *Proceedings of the 20th ACM International Conference on Information and Knowledge Management*, 423–432 (2011).

[4] De Felipe, I., Hristidis, V. & Rishe, N. Keyword search on spatial databases. In *2008 IEEE 24th International Conference on Data Engineering,* 656–665 (2008).

[5] Khodaei, A., Shahabi, C. & Li, C. Hybrid indexing and seamless ranking of spatial and textual features of web documents. In *International Conference on Database and Expert Systems Applications*, 450–466 (2010).

[6] Li Z, Lee KCK, Zheng B, Lee WC (2011). IR-tree: An efficient index for geographic document search. IEEE Trans. Knowl. Data Eng..

[7] Vaid, S., Jones, C.B., Joho, H. & Sanderson, M. Spatio-Textual indexing for geographical search on the web. In *International Symposium on Spatial and Temporal Databases*, 218–235 (2005).

[8] Zhang C, Zhang Y, Zhang W, Lin X (2016). Inverted linear quadtree: Efficient top k spatial keyword search. IEEE Trans. Knowl. Data Eng..

[9] Wu, D., Li, Y., Choi, B. & Xu, J. Social-aware top-k spatial keyword search. In *2014 IEEE 15th International Conference on Mobile Data Management*, 14–18 July 2014 Brisbane. QLD: IEEE, 1, 235–244 (2014).

[10] Ahmed, P., Hasan, M., Kashyap, A., Hristidis, V. & Tsotras, V.J. Efficient computation of top-k frequent terms over spatio-temporal ranges. In *Proceedings of the 2017 ACM International Conference on Management of Data*. 1227–1241 (2017).

[11] Qian Z, Xu J, Zheng K, Zhao P, Zhou X (2018). Semantic-aware top-k spatial keyword queries. World Wide Web.

[12] Attique M, Afzal M, Ali F, Mehmood I, Ijaz MF, Cho HJ (2020). Geo-social top-k and skyline keyword queries on road networks. Sensors.

[13] Chen X, Xu J, Zhou R, Zhao P (2020). S2R-tree: A pivot-based indexing structure for semantic-aware spatial keyword search. GeoInformatica.

[14] Kwon HY, Whang KY, Song IY, Wang H (2013). RASIM: A rank-aware separate index method for answering top-k spatial keyword queries. World Wide Web.

[15] Guttman, A. R-trees: A dynamic index structure for spatial searching. In *Proceedings of the 1984 ACM SIGMOD International Conference on Management of Data*, June 1984 New York. NY: Association for Computing Machinery, 47–57 (1984).

[16] Wille, R. Restructuring lattice theory: An approach based on hierarchies of concepts. In: Rival I, (eds) Ordered Sets. NATO Advanced Study Institutes Series (Series C — Mathematical and Physical Sciences), vol 83, 445–470 (1982).

[17] Agrawal, R. & Srikant, R. Fast algorithms for mining association rules. In *Proc. 20th Int. Conf. Very Large Data Bases*, September 1994 Santiago, 1215: 487–499 (1994).

[18] Han J, Pei J, Yin Y (2000). Mining frequent patterns without candidate generation. ACM SIGMOD Rec..

[19] Li X, Lin H (2007). Indexing network-constrained trajectories for connectivity-based queries. Int. J. Geogr. Inf. Sci..

[20] Rahimi M, Malek MR, Claramunt C (2021). A topology-based graph data model for indoor spatial-social networking. Int. J. Geogr. Inf. Sci..

[21] Rocha-Junior, J. B. & Nørvåg, K. Top-k spatial keyword queries on road networks. In T*he 15th International Conference on Extending Database Technology (EDBT '12)*. Association for Computing Machinery, New York, NY, USA, 168–179 (2012).

[22] Attique, M., Cho, H. J. & Chung, T. S. Efficient processing of moving top- k spatial keyword queries in directed and dynamic road networks. *Wirel. Commun. Mobile Comput.* 1–19 (2018).

[23] Finkel RA, Bentley JL (1974). Quad trees A data structure for retrieval on composite keys. Acta Informatica.

[24] Xu T, Zhang X, Claramunt C, Li X (2018). TripCube: A Trip-oriented vehicle trajectory data indexing structure. Comput. Environ. Urban Syst..

[25] Chen, Y. Y., Suel, T. & Markowetz, A. Efficient query processing in geographic web search engines. In *The 2006 ACM SIGMOD international conference on Management of data*, 277–288 (2006).

[26] Chen, J., Xu, J., Liu, C., Li, Z. & Ding, Z. Multi-objective spatial keyword query with semantics. In *International Conference on Database Systems for Advanced Applications*, 34–48 (2017).

[27] Sun, J., Xu, J., Zheng, K. & Liu, C. Interactive spatial keyword querying with semantics. In *Proceedings of the 2017 ACM on Conference on Information and Knowledge Management*, 1727–1736 (2017).

[28] Xu D, Tian Z, Lai R, Kong X, Tan Z, Shi W (2020). Deep learning based emotional analysis of microblog texts. Inf. Fusion.

[29] Shafiq M, Tian Z, Bashir A, Jolfaei A, Yu X (2020). Data mining and machine learning methods for sustainable smart cities traffic classification: A survey. Sustain. Cities Soc..

[30] Tian Z, Luo C, Lu H, Su S, Sun Y, Zhang M (2020). User and entity behavior analysis under urban big data. ACM/IMS Trans. Data Sci..

[31] Zhao X, Zhang Z, Huang H, Bi X (2020). Social-aware spatial keyword top-k group query. Distrib. Parallel Databases.

[32] Sohail A, Cheema MA, Taniar D (2018). Social-aware spatial top-k and skyline queries. Comput. J..

[33] Eldawy A. & Mokbel M. F. The era of big spatial data. *2016 IEEE 32nd International Conference on Data Engineering (ICDE)*, 1424–1427, 10.1109/ICDE.2016.7498361 (2016).

[34] Xu J, Sun J, Zhou R, Liu C, Yin L (2021). CISK: An interactive framework for conceptual inference based spatial keyword query. Neurocomputing.

[35] Schwering, A. & Raubal, M. Spatial relations for semantic similarity measurement. *Lecture Notes in Computer Science*, *International Conference on Conceptual Modeling, ER 2005*, vol 3770, 259–269 (2005).

[36] Kainz W, Egenhofer MJ, Greasley I (1993). Modelling spatial relations and operations with partially ordered sets. Int. J. Geogr. Inf. Syst..

[37] Chen, J., Huang, F., Wang, R., et al. A Research about spatial association rule mining based on concept lattice. In *International Conference on Wireless Communications, Networking and Mobile Computing*. 21–25 September 2007 Shanghai: IEEE, 2007: 5979–5982 (2007).

[38] Tripathy, A., Mishra, L. & Patra P. K. A multi dimensional design framework for querying spatial data using concept lattice. In *2010 IEEE 2nd International Advance Computing Conference (IACC)*. 19–20 February 2010 Patiala: IEEE, 394–399 (2007).

[39] Wu X, Wang J, Shi L (2019). A fuzzy formal concept analysis-based approach to uncovering spatial hierarchies among vague places extracted from user-generated data. Int. J. Geogr. Inf. Sci..

[40] Xu H, Wang C, Dong K, Yue Z (2019). Identification and prediction of interdisciplinary research topics: A study based on the concept lattice theory. J. Data Inf. Sci..

[41] Sampath S, Sprenkle S, Gibson E, Pollock L, Greenwald AS (2007). Applying concept analysis to user-session-based testing of web applications. IEEE Trans. Softw. Eng..

[42] Zou C, Zhang D, Wan J, Hassan MM, Lloret J (2015). Using concept lattice for personalized recommendation system design. IEEE Syst. J..

[43] Nguyen PHP, Corbett D (2005). A basic mathematical framework for conceptual graphs. IEEE Trans. Knowl. Data Eng..

